# Timing of IABP initiation and its impact on outcomes in acute myocardial infarction with cardiogenic shock: insights from a bi-center retrospective study

**DOI:** 10.1007/s00392-026-02951-1

**Published:** 2026-06-08

**Authors:** Istvan Bojti, Sarolta Bojtine Kovacs, David Kovacs, Antonia Ziegler, Alexander Maier, Dirk Westermann, Miroslaw Ferenc, Attila Csaba Nagy, Kalman Racz, Zsolt Koszegi, Gabor Tamas Szabo

**Affiliations:** 1 Department of Cardiology and Angiology, Faculty of Medicine, University Heart Center Freiburg, University of Freiburg, Freiburg, Germany; 2 IMM-PACT Cluster of Excellence, Faculty of Medicine, University of Freiburg, Freiburg, Germany; 3 Section of Molecular Hematology, Department of Medicine I, Hematology, Oncology and Stem Cell Transplantation, Medical Center, Faculty of Medicine, University of Freiburg, University of Freiburg, Freiburg, Germany; 4 Department of Otorhinolaryngology and Head-Neck Surgery, Faculty of Medicine, University of Debrecen, Debrecen, Hungary; 5 Department of Epidemiology, Faculty of Health Sciences, University of Debrecen, Debrecen, Hungary; 6 Department of Forensic Medicine, Faculty of Medicine, University of Debrecen, Debrecen, Hungary; 7 Department of Cardiology and Cardiac Surgery, Faculty of Medicine, University of Debrecen, Debrecen, Hungary

**Keywords:** Acute myocardial infarction, Cardiogenic shock, IABP, Area at risk

## Abstract

**Background:**

The optimal timing of intra-aortic balloon pump (IABP) initiation in cardiogenic shock (CS) complicating acute myocardial infarction (AMI) remains uncertain. Contemporary ESC guidance and the recent EACTS/STS/AATS MCS guideline highlight gaps regarding timing, patient selection, and ischemic burden. In this study, the association between IABP timing, myocardial area at risk (AAR), and survival in AMI-CS was evaluated.

**Methods:**

We retrospectively analyzed 399 AMI-CS patients treated with primary PCI at two tertiary centers. Patients were categorized as no IABP (*n* = 124), non-rescue IABP (started during PCI; *n* = 216), or rescue IABP (inserted after PCI; *n* = 59). Clinical and angiographic parameters, including AAR quantified by a coronary anatomy–based algorithm, were assessed. Multivariable logistic regression identified predictors of in-hospital and 1-year mortality.

**Results:**

Non-rescue IABP was independently associated with lower in-hospital mortality vs. no IABP (OR 0.29, 95% CI 0.16–0.52). Rescue IABP showed a weaker association with lower in-hospital mortality (OR 0.41, 95% CI 0.19–0.89). At 1 year, mortality was higher in both no IABP (OR 3.27, 95% CI 1.86–5.76) and rescue IABP groups (OR 2.04, 95% CI 1.04–3.98) compared with non-rescue IABP. An augmented inverse-probability weighted (AIPW) analysis confirmed these findings, demonstrating a significant reduction in 1-year mortality with early IABP use compared with no IABP (ATE − 0.24, 95% CI − 0.375 to − 0.104), whereas no significant effect was observed for rescue IABP. A significant interaction between AAR and timing indicated that early IABP offered the greatest benefit in patients with moderate AAR (30–55%), whereas no benefit was observed with extensive AAR (> 80%).

**Conclusions:**

In AMI-CS, early IABP initiation was associated with improved in-hospital and long-term survival, particularly in patients with a moderate AAR. These findings support the need for refining IABP use based on both ischemic burden and the timing of hemodynamic support.

**Trial registration number and date of registration.:**

DRKS00038637, 03.12.2025.

**Graphical Abstract:**

**Figure:**
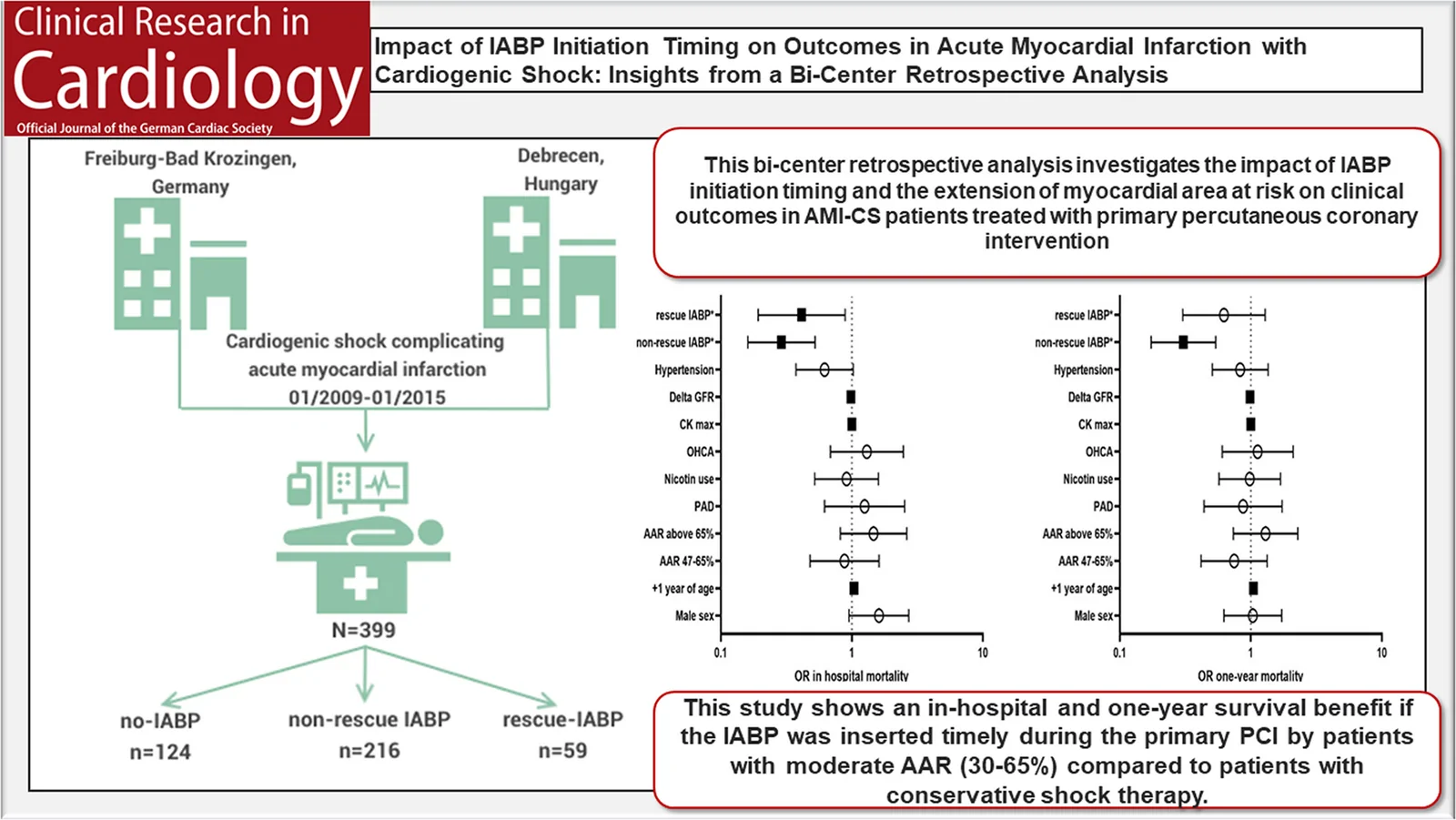

**Supplementary Information:**

The online version contains supplementary material available at https://doi.org/10.1007/s00392-026-02951-1.

## Introduction

Cardiogenic shock (CS) represents a heterogeneous clinical syndrome arising from multiple etiologies—including acute myocardial infarction (AMI), acute decompensated heart failure, fulminant myocarditis, valvular disease, and profound post-cardiac surgery failure—and remains associated with mortality rates exceeding 40% despite significant advances in revascularization and intensive care [1]. Regardless of its underlying etiology, CS is characterized by rapidly progressive circulatory collapse and end-organ hypoperfusion, often emerging mechanical circulatory support (MCS) devices to stabilize hemodynamics and bridge patients to recovery or advanced therapies [2, 3]. MCS modalities provide varying levels of circulatory augmentation, the intra-aortic balloon pump (IABP) offers modest support (0.5–1.0 L/min), while the Impella system provides more substantial cardiac unloading (2.5–4.0 L/min) [4], and veno-arterial extracorporeal membrane oxygenation (VA-ECMO) delivers full cardiopulmonary support but requires extensive expertise and resources [5]. Observational data suggest that early MCS initiation—particularly before irreversible end-organ damage—may improve survival across CS etiologies [6, 7].

The recently published EACTS/STS/AATS guideline on temporary mechanical circulatory support, focusing on short-term MCS use in adult cardiac surgery and perioperative cardiogenic shock, highlights persistent gaps in the evidence base for AMI-related CS (AMI-CS). Specifically, the guideline emphasizes that the most relevant randomized trial—IABP-SHOCK II—demonstrated no mortality benefit with routine IABP use in AMI-CS, yet did not evaluate the timing of IABP initiation or stratify key subgroups, including patients with varying degrees of threatened myocardium [8, 9]. Similar concerns were raised by Szabó et al. regarding the BCIS-1 study evaluating IABP use in high-risk PCI, in which neither the extent of jeopardized myocardium nor the precise timing of counterpulsation initiation were prospectively stratified, potentially contributing to the trial’s neutral results [10, 11]. These limitations reinforce key unanswered questions, particularly which CS patients and at what stage of ischemic injury may benefit most from IABP support?

Notably, the concept of a jeopardized myocardial territory—commonly expressed as the area at risk (AAR)—is relevant not only in AMI-CS but across multiple CS contexts. In AMI-CS, the AAR represents acutely ischemic but potentially salvageable myocardium whose reperfusion and unloading can prevent infarct expansion, left ventricular dysfunction, and refractory shock [12]. Even in selected forms of non-ischemic CS, such as acute myocarditis or decompensated heart failure, the amount of salvageable myocardial tissue—or the degree of metabolic and microvascular reserve—may influence the effectiveness of early support [13]. Thus, across etiologies, the presence and extent of jeopardized but viable myocardium may determine both the biological plausibility and potential impact of MCS therapies. Despite its modest hemodynamic augmentation, IABP remains the most accessible and affordable temporary MCS modality worldwide. Its relatively low cost renders it especially important in low- and middle-income countries (LMICs), where advanced devices such as Impella or VA-ECMO are often unavailable due to infrastructure limitations [14].

While current guidelines on AMI-CS recommend IABP for mechanical complications or as a bridge to advanced therapies [15], the evidence-based timing and more detailed patient selection remain insufficient. Addressing this question, an ongoing RCT investigates the effect of an early IABP initiation in non-ischemic CS [16].

Given the heterogeneity of CS, the emerging recognition of AAR as a central determinant of therapeutic effectiveness across etiologies, and the persistent uncertainty regarding when and for whom IABP may offer benefit, further investigation is warranted. This bi-center retrospective analysis investigates the impact of IABP initiation timing and the extension of myocardial area at risk on clinical outcomes in AMI-CS patients treated with primary percutaneous coronary intervention (pPCI).

## Methods

Patients presenting with AMI complicated by CS who underwent a successful pPCI (TIMI flow ≥ 2) between January 2009 and January 2015 at two tertiary care heart centers, Department of Cardiology and Heart Surgery, University of Debrecen (Hungary) and University Heart Center Freiburg-Bad Krozingen (Germany), were screened. Inclusion criteria consisted of patients aged ≥ 18 years with a diagnosis of AMI [17] and CS at admission, defined as systolic blood pressure < 90 mmHg for ≥ 30 min, signs of end-organ hypoperfusion, or the need for inotropes or vasopressors [18], all of whom were treated with pPCI and fluid challenge, and vasopressor/inotropic and respiratory failure were managed according to the guidelines in effect [17, 18]. Patients were excluded if they underwent surgical revascularization (aorto-coronary bypass graft operation (ACBG)), or a mechanical complication was diagnosed by echocardiographic examination at admission or had inconclusive or missing medical data.

### Data collection

Medical, laboratory, and pPCI procedural data were extracted from electronic health records. Collected variables included demographics (age, sex, comorbidities), hemodynamic parameters (blood pressure, heart rate, lactate levels), laboratory markers (peak creatine kinase [CK], glomerular filtration rate [GFR] and changes, hemoglobin and changes), pPCI procedural details (culprit vessel, TIMI flow post-PCI), adverse events during hospitalization (arterial punction site-related complication, infection, bleeding event, allergic reaction, ACS-related mechanical complication), and outcomes (in-hospital mortality rate, 1-year survival rate).

### Area at risk determination

The myocardial area at risk (AAR) was calculated using an algorithm based on coronary anatomy and angiographic culprit lesion localization, as previously described [19, 20]. This method integrates coronary dominance (right, left, balanced, or super right), length of the LAD, and culprit lesion location to generate a reconstruction of the jeopardized myocardium. AAR was expressed as a percentage of the affected left ventricular segments of the 17 LV segments model recommended by the AHA [21].

### Statistical analysis

Descriptive statistics were used to summarize baseline characteristics. Continuous variables were expressed as median (interquartile range [IQR]) and compared using the Mann–Whitney *U* test or Kruskal–Wallis test, while categorical variables were expressed as frequencies (%) and compared using the Pearson *χ*^2^ test or Fisher’s exact test. To identify thresholds with potential prognostic relevance, receiver operating characteristic (ROC) analysis was performed using in-hospital and 1-year mortality as dependent variables. These thresholds were subsequently applied to define categorical AAR groups for inclusion in the multivariable logistic regression models. To identify predictors of in-hospital and 1-year mortality, univariate logistic regression analyses were first performed for all candidate variables. Variables associated with outcomes in univariate analyses were considered for multivariable modeling. In addition, clinically and historically relevant covariates were included in the multivariable models regardless of their univariate statistical significance, based on prior evidence and pathophysiological considerations, to account for potential confounding. These covariates included age, sex, hypertension, peripheral artery disease (PAD), nicotine use, prehospital resuscitation, AAR categories, peak creatine kinase (CK), and change in glomerular filtration rate (GFR) during hospitalization.

Multivariable logistic regression was then performed to identify independent predictors of in-hospital and 1-year mortality. Odds ratios (OR) with 95% confidence intervals (CI) are reported, and a two-sided *p* value < 0.05 was considered statistically significant. Subgroup analyses compared outcomes between no IABP versus non-rescue IABP, no IABP versus rescue IABP, and non-rescue versus rescue IABP. To account for potential confounding by indication and to address baseline imbalances between the study groups, an augmented inverse probability weighting (AIPW) analysis was performed. The average treatment effect (ATE) on 1-year mortality was estimated. The treatment model (a multinomial logit for the primary three-group comparison, and a binary logit for sensitivity analyses) and the outcome model (linear) were adjusted for clinically relevant covariates: age, sex, categorical area at risk (AAR), peripheral artery disease (PAD), and smoking status. Covariate balance before and after weighting was assessed using standardized differences and variance ratios, with a standardized difference of < 0.1 considered indicative of good balance. Visual inspection of propensity score overlap and covariate density plots was additionally performed to ensure adequate common support. All analyses were conducted using Intercooled Stata version 17.

## Results

Between January 2009 and January 2015, a total of 399 patients presenting with acute myocardial infarction complicated by cardiogenic shock met the inclusion criteria and did not fulfill any exclusion criteria across the two participating tertiary centers. Patients were categorized into three groups: patients without IABP treatment (no IABP; *n* = 124), patients received IABP during pPCI (non-rescue IABP; *n* = 216), and patients where IABP was initiated after pPCI during hospitalization (rescue IABP; *n* = 59).

### Baseline characteristics

#### Non-rescue vs. rescue IABP

In the analysis of patients receiving IABP support, outcomes were compared between those who received IABP in the same setting of the pPCI (non-rescue, *n* = 216) and those who received IABP later during hospitalization (rescue, *n* = 59), with a dealy of time between pPCI and IABP insertion of 50 (SD: 53) hours. Baseline characteristics, including age, gender, prevalence of diabetes, hypertension, peripheral artery disease (PAD), and nicotine use, were similar between groups (Table 1).

**Table 1:** Baseline characteristics. Values are presented as median (interquartile range [IQR]) or number (percentage), as appropriate. Comparisons between groups were performed using the Mann–Whitney *U* test or Kruskal–Wallis test for continuous variables, and the Pearson *χ*^*2*^ test or Fisher’s exact test for categorical variables. ΔGFR indicates the change in glomerular filtration rate (GFR) during hospitalization; AAR, area at risk; CK, creatine kinase; GFR, glomerular filtration rate; IABP, intra-aortic balloon pump; IQR, interquartile range; LV, left ventricle; OHCA, out-of-hospital cardiac arrest; PAD, peripheral artery disease

	(1) No IABP *n* = 124	(2) Non-rescue IABP *n* = 216	(3) Rescue IABP *n* = 59	*P*		
				1 vs 2	1 vs 3	2 vs 3
Male *n* (%)	88 (71)	135 (62,5)	38 (64.4)	0.114	0.370	0.788
Age years (IQR)	72.5 (65–80)	66 (57–74)	68 (59–75)	** < 0.001**	**0.002**	0.822
Diabetes mellitus *n* (%)	40 (32.3)	75 (34.7)	17 (28.8)	0.644	0.638	0.394
Hypertension *n* (%)	81 (65.3)	138 (63.9)	39 (66.1)	0.790	0.917	0.753
PAD *n* (%)	6 (4.8)	40 (18.5)	9 (15.3)	** < 0.001**	**0.016**	0.561
Nicotin use *n* (%)	51 (41.1)	66 (31)	24 (41.4)	0.059	0.975	0.136
OHCA *n* (%)	34 (27.4)	51 (23.6)	5 (8.5)	0.435	**0.003**	**0.011**
AAR *%* (IQR)	53 (35–76)	59 (47–88)	53 (41–82)	**0.008**	0.680	0.075
CK max U/L (IQR)	328.5 (121.5–655.5)	1275 (288–3749)	904 (321–2748)	** < 0.001**	** < 0.001**	0.299
GFR at admission *ml/min/1.73 m*^*2*^ (IQR)	57 (42–77.5)	60 (40–82)	77 (52–90)	0.908	**0.003**	**0.001**
GFR control ml/min/1.73 m^2^ (IQR)	49 (33–79)	55 (31–83)	56 (32–90)	0.882	0.445	0.509
ΔGFR *ml/min/1.73 m*^*2*^ (IQR)	− 7 (− 14–8)	− 1 (− 15–5)	− 9 (− 26–0)	0.525	0.065	**0.017**
Hemoglobin *g/l* (IQR)	136 (125.5–149)	137 (123–147)	138 (123–154)	0.644	0.575	0.337
Thrombocytes ×10⁹/L (IQR)	226.5 (186–276)	246 (202–297)	244.5 (210–305)	**0.031**	0.097	0.926

The area at risk (AAR) was numerically higher in the non-rescue group (59% vs. 53%, *p* = 0.075), though not statistically significant, similar to peak creatine kinase (CK) levels (1275 U/L vs. 904 U/L, *p* = 0.299). Baseline renal function was significantly better in the rescue group (GFR 60 vs. 77 mL/min/1.73 m^2^, *p* = 0.001), though follow-up GFR and change in GFR (ΔGFR) showed greater deterioration in the rescue group (ΔGFR − 1 vs. − 9 mL/min, *p* = 0.017). Prehospital resuscitation rates were significantly lower in the rescue group (8.5% vs. 23.6%, *p* = 0.011). Complication rates were comparable between groups, including bleeding, site complications, infections, and mechanical complications (Tables 1 and 2).

**Table 2:** Complications. Values are presented as *number (percentage)* unless otherwise indicated. Comparisons between groups were performed using the *χ*^2^ test or Fisher’s exact test, as appropriate

	(1) No IABP *n* = 124	(2) Non-rescue *n* = 216	(3) Rescue *n* = 59	*P*		
				1 vs 2	1 vs 3	2 vs 3
Bleeding *n* (%)	4 (3.2)	10 (4.6)	5 (8.5)	0.531	0.125	0.249
Punction site complication *n* (%)	3 (2.4)	14 (6.5)	2 (3.4)	0.098	0.707	0.369
Infection *n* (%)	40 (32.3)	73 (33.8)	21 (35,6)	0.772	0.655	0.796
Mechanical complication *n* (%)	3 (2.4)	3 (1.4)	3 (5.1)	0.487	0.344	0.085

#### No IABP vs. non-rescue IABP

Peak CK levels were substantially higher in the non-rescue IABP group (1275 U/L vs. 328.5, *p* < 0.001). Thrombocyte count on admission was significantly higher in the non-rescue IABP group compared to the no IABP, but both values were within the normal range. Area at risk (AAR) has been calculated significantly higher in the non-rescue IABP group (59% vs. 53%, *p* = 0.008). The history of peripheral artery disease (PAD) was more prevalent in the non-rescue IABP group (18.5% vs. 4.8%, *p* > 0.001). Prehospital resuscitation rates did not differ significantly in the two groups.

#### No IABP vs. rescue IABP

Peak CK levels were markedly higher in the rescue IABP group (904 vs. 328.5 U/L, *p* < 0.001). Baseline GFR was better in the rescue IABP group (57 vs. 77.0 mL/min, *p* = 0.003), but renal function deterioration (ΔGFR) was similar (*p* = 0.065). Peripheral artery disease (PAD) was more prevalent in the rescue IABP group (15.3% vs. 4.8%, *p* = 0.016). Prehospital resuscitation rates were significantly lower in the rescue IABP group compared to the no IABP patients (8.5% vs. 27.4%, *p* = 0.003) (Table 1).

### Procedural outcomes

Complication rates, including bleeding, punction site-related complications, infections, allergic reaction, and mechanical complications, did not differ significantly among the groups (Table 2).

### Survival analysis

**Fig. 1:**
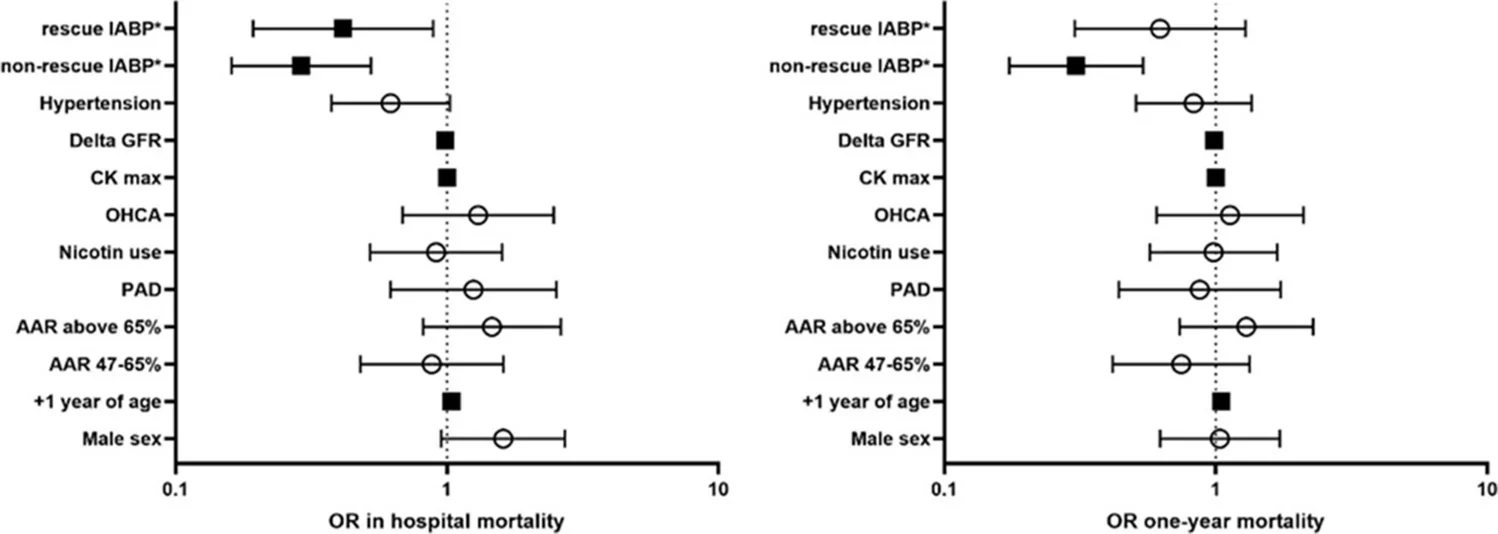
Forest plot illustrating the results of the multivariable logistic regression analysis for in-hospital (left) and 1-year (right) mortality in patients with acute myocardial infarction-related cardiogenic shock. Odds ratios (OR) and 95% confidence intervals (CI) are displayed for each variable included in the final model. Variables entered into the analysis included IABP timing (non-rescue vs rescue), AAR (area at risk) category (47–65% and > 65%), age, sex, hypertension, peripheral artery disease (PAD), nicotin abusus, prehospital resuscitation (OHCA), peak CK, and GFR change during hospitalisation. Significant predictors are displayed as black boxes and non-significant variables as open circles; horizontal lines represent 95% CI. The vertical dashed line denotes the line of no effect (OR = 1). *Comparisons versus the no IABP group

After establishing the baseline characteristics of the study population, we next examined the associations between these variables and the in-hospital and 1-year survival using logistic regression analysis to identify key predictors while adjusting for potential confounders (Table 3). Receiver operating characteristic (ROC) analysis identified two AAR intervals—47–65% and > 65% of left ventricular involvement—which demonstrated the highest, though modest, sensitivity and specificity for in-hospital and 1-year mortality prediction. These cut-off values were therefore used to define categorical AAR groups for subsequent analyses. Results of the regression analysis are summarized in Fig. 1.

**Table 3:** Survival data. Values are expressed as number (percentage) unless otherwise indicated. In-hospital and 1-year survival was compared across groups using the *χ*^2^ test

	(1) No IABP *n* = 124	(2) Non-rescue *n* = 216	(3) Rescue *n* = 59	*P*		
				1 vs 2	1 vs 3	2 vs 3
Hospital mortality *n* (%)	62 (50)	71 (32.9)	20 (33.9)	**0.002**	**0.041**	0.882
1-year mortality *n* (%)	73 (58.9)	80 (37)	30 (50.9)	** < 0.001**	0.306	0.055

#### In-hospital survival

Patients managed with non-rescue IABP support had an OR of 0.29 (95% CI 0.16–0.52, *p* < 0.001), patients with rescue IABP had an OR of 0.41 (95% CI 0.19–0.89, *p* = 0.024) of in-hospital mortality compared to those receiving no IABP. When comparing non-rescue IABP patients with rescue IABP patients, latter group had an OR of 1.42 (95% CI 0.7–2.91, *p* = 0.328) for in-hospital mortality. Each unit increase in peak CK was associated with a slight but significant increase (OR = 1.00017 (95% CI 1.00008–1.00027, *p* < 0.001)) in mortality risk. The lower deterioration of GFR by each 1 mL/min/1.73m^2^ was associated with a 1.5% mortality risk reduction (OR = 0.99 (95% CI 0.972–0.99, *p* = 0.042)). Higher age led to higher in hospital mortality; OR = 1.04 (95% CI 1.014–1.067, *p* = 0.002). Gender, AAR, nicotine use, prehospital resuscitation, hypertension, and peripheral artery disease did not show statistically significant associations with this outcome.

#### One-year survival

Patients managed with non-rescue IABP had an OR of 0.31 (95% CI 0.17–0.54, *p* < 0.001); patients with rescue IABP had an OR of 0.62 (95% CI 0.3–1.28, *p* = 0.2) of 1-year all cause mortality compared to those receiving no IABP. When comparing non-rescue IABP patients with rescue IABP patients, latter group had an OR of 2.04 (95% CI 1.04–3.98, *p* = 0.037) for this endpoint. Each unit increase of the peak creatine kinase (CKmax, OR 1.0002, 95% CI 1.00007–1.0002, *p* = 0.001) and each 1 mL/min/1.73m^2^ more drop of GFR during the hospitalisation (OR 0.986, 95% CI 0.97–0.99, *p* = 0.038) were significantly associated with 1-year mortality risk. Each year age gap was associated with an OR = 1.05 (95% CI 1.02–1.07, *p* < 0.001) in 1-year mortality. Gender, AAR, smoking status, prehospital resuscitation, hypertension, and peripheral artery disease did not show statistically significant associations with this outcome.

## Augmented inverse-probability weighted analysis of IABP timing and outcomes

Before weighting, baseline characteristics exhibited significant imbalances between the treatment and control groups. Following AIPW adjustment, covariate balance improved substantially, with standardized differences < 0.1 for most covariates; residual imbalance persisted for PAD in the rescue-IABP comparison (Supplementary Table 1), and sufficient common support was confirmed by overlap plots (Supplementary Fig. 1).

The AIPW analysis demonstrated a significant reduction in the probability of 1-year mortality associated with early IABP use. Specifically, the average treatment effect (ATE) for early IABP versus no IABP was − 0.24 (95% CI − 0.375 to − 0.104; *p* = 0.001), corresponding to a 24 percentage point absolute risk reduction. In contrast, rescue IABP therapy did not show a statistically significant survival benefit compared to the control group (ATE = − 0.13, 95% CI − 0.300 to 0.040; *p* = 0.136).

A sensitivity analysis excluding the rescue-IABP cohort yielded highly consistent results for early IABP use compared to controls (ATE = − 0.24, 95% CI − 0.380 to − 0.096; *p* = 0.001). Finally, when evaluating IABP therapy overall—without distinguishing by the timing of insertion—the ATE on one-year mortality was − 0.18 (95% CI − 0.300 to − 0.066; *p* = 0.002). These propensity-based results robustly align with our multivariable regression models, reinforcing the hypothesis that the timing of IABP insertion is a critical modifier of its clinical efficacy.

## Stratified analysis: the impact of area at risk and the timing of IABP initiation on mortality

A significant interaction was observed between the initiation of IABP treatment and the extent of AAR (*p* = 0.019) in relation to in-hospital mortality. Specifically, IABP initiation during the pPCI (non-rescue group) demonstrated the most pronounced protective effect against in-hospital mortality in patients with a moderate AAR (30–55%). However, this protective effect decreased continuously with the elevation of AAR percentage and was no longer observed in patients with an AAR > 80%. In contrast, IABP in patients with delayed initiation (rescue IABP group) did not show a significant association with AAR in terms of survival outcomes.

In the non-rescue IABP group, patients who were deceased during hospitalization had a significantly larger AAR compared to those in the the no IABP and rescue IABP groups (71% vs. 53% vs. 47%, respectively; *p* < 0.002 and *p* = 0.005). This trend persisted in the extended analysis including patients who died within the first-year after CS. In this analysis, the non-rescue IABP group again exhibited the highest AAR compared to the no IABP and rescue-IABP groups (71% vs. 53% vs. 47%, respectively; *p* = 0.013 and *p* = 0.019). When comparing the AAR of the surviving and deceased patients in the 3 groups, only the non-rescue IABP treatment shows a significant difference with higher AAR in case of the deceased patients (Table 4).

**Table 4:** Area at risk according to treatment groups and mortality. Values are expressed as median (interquartile range [IQR]) unless otherwise indicated. Patients were stratified by IABP initiation timing-no IABP, non-rescue IABP (inserted during primary percutaneous coronary intervention [pPCI]), and rescue IABP (inserted after pPCI)-and by outcome (survivor vs. deceased) for both in-hospital and 1-year follow-up. The myocardial area at risk (AAR) was calculated using a validated angiographic algorithm integrating coronary dominance, left anterior descending artery (LAD) length, and culprit lesion localization, expressed as a percentage of the total left ventricular (LV) myocardial mass. Comparisons between groups were performed using the Mann–Whitney *U* test

In-hospital mortality	Group	Survivor	Deceased	*p*-value
Area at risk *%* (Mdn IQR)	No-IABP	53 (35–82)	53 (35–71)	0.986
	Non-rescue IABP	53 (47–76)	71 (53–88)	**0.003**
	Rescue IABP	53 (41–88)	47 (38–68)	0.234
1-year mortality	Group	Survivor	Deceased	*p*-value
Area at risk *%* (Mdn IQR)	No-IABP	47 (35–76)	53 (35–76)	0.497
	Non-rescue IABP	53 (47–76)	71 (50–88)	**0.009**
	Rescue IABP	53 (41–76)	47 (35–88)	0.464

## Discussion

This study addresses the evidence gap, evaluating the impact of IABP timing and extent of infarction area at risk (AAR) in AMI-CS population. In our bi-center retrospective analysis, we demonstrated that IABP initiation at the time of pPCI (non-rescue) by patients with moderate area at risk (30–65%) is associated with significantly improved in-hospital and 1-year survival in AMI-CS patients compared to patients with delayed insertion of IABP (rescue) or treated without MCS (no IABP). Acknowleding the inherent limitation of our study, particularly the retrospective and non-randomized design, the present findings should be interpreted as hypothesis-generating and associative, rather than establishing causality.

The IABP-SHOCK II trial—the most relevant randomized study in this patient population—could not demonstrate survival benefit of the IABP treatment; however, the trial did not aim to stratify patients by timing of IABP initiation or the extension of the jeopardized myocardium (AAR). In our analysis, we were focusing on these unanswered questions and trying to characterize an AMI-CS patient population with benefit most from IABP treatment. Our retrospective analysis suggests that the timing of IABP and the extension of the jeopardized myocardium in AMI (AAR) should be considered, particularly, early placement of IABP (at the time of PCI) in patients with an AAR between 30 and 65% may confer a survival advantage. Importantly, our findings do not contradict IABP-SHOCK II but rather suggest that timing of support and ischemic burden may represent clinically relevant effect modifiers that were not prospectively evaluated in prior randomized trials. Accordingly, our results should be viewed as contributing to the ongoing discussion on selective IABP use, particularly in settings where advanced MCS is not readily available.

Several mechanisms may explain the observed benefits of early IABP initiation. First, IABP reduces afterload and myocardial oxygen demand, thereby improving coronary perfusion and limiting infarct expansion [7]. Second, by augmenting diastolic pressure, IABP may enhance microvascular perfusion and reduce ischemia–reperfusion injury [12]. Third, early hemodynamic stabilization may mitigate multi-organ failure, particularly renal dysfunction, which is a strong predictor of mortality in CS [22]. Although these mechanisms may also operate when IABP is initiated later in the clinical course, their effectiveness likely diminishes as irreversible myocardial and systemic damage progress. Once significant infarct expansion, microvascular obstruction, or end-organ hypoperfusion have already developed, mechanical support may not fully reverse these pathophysiological cascades. The present findings therefore suggest that timely IABP initiation, before extensive tissue injury and metabolic derangement occur, is critical to maximize hemodynamic and myocardial benefits. In this context, early intervention appears not merely supportive but potentially preventive in attenuating the downward spiral of cardiogenic shock [23]. It should be mentioned here that these pathophysiological considerations should be interpreted first with caution, as the observed associations may also reflect differences in patient selection and timing of intervention rather than a direct treatment effect.

Despite more pronounced AAR and comparable baseline comorbidities, patients treated with IABP had significantly better short- and long-term survival. The IABP group was younger but had a higher prevalence of peripheral artery disease which may reflect selection bias, with IABP being used in patients perceived to have a better prognosis or greater hemodynamic reserve. The higher in-hospital survival (ca. 65%) in our IABP population mirrors the observation of a recent, large, nationwide retrospective comparative analysis of approximately 65.000 patients [24]. Notably, peak CK levels were higher in the IABP group despite improved outcomes. Although this finding may reflect residual confounding, it also raises the possibility that early IABP support may mitigate the clinical impact of larger infarcts, suggesting a potential interaction between infarct size and timing of mechanical support. The similar complication rates between groups suggest that IABP did not confer additional procedural risks while being associated with improved survival in this cohort.

Non-rescue IABP placement was associated with a trend toward improved 1-year survival compared to delayed (rescue) IABP placement and at the same time higher prehospital resuscitation rates, suggesting that these patients may have presented with more severe initial clinical status. The rescue group had better baseline renal function but experienced greater renal deterioration during hospitalization, potentially reflecting delayed hemodynamic stabilization. This observation is in line with recent observations of Yan et al. [25]. Importantly, the definition of rescue IABP does not allow clear differentiation between delayed treatment and a more advanced disease phenotype, which limits interpretation of timing effects.

No IABP was associated with a 4.32-times higher in-hospital mortality risk compared to non-rescue IABP, reinforcing the importance of early mechanical circulatory support. Higher peak CK (representing larger infarcts and more impacted renal function) and worsening follow-up GFR value (renal deterioration) were independent predictors of mortality, emphasizing also the need and effectiveness of early circulatory support and renal protection. These findings were further supported by an augmented inverse-probability weighted (AIPW) analysis, which confirmed the association between early IABP use and lower 1-year mortality while preserving the full cohort and improving adjustment for baseline differences. Nevertheless, residual confounding cannot be excluded, particularly due to unmeasured variables such as shock severity.

The observed protective effect of early IABP initiation in patients with a moderate AAR (30–65%) aligns with the hypothesis that mechanical circulatory support may mitigate ischemia–reperfusion injury and improve microvascular perfusion, but this effect is limited by the extension of myocardial ischemia and in regions where salvageable myocardium is still present [26]. In these patients, IABP-induced augmentation of coronary perfusion and reduction of afterload may limit infarct expansion and support left ventricular function. However, the absence of benefit in patients with an extended AAR (> 80%) could reflect that the augmentation by counterpulsation is not sufficient. Together, these findings support a more targeted, physiology-guided approach to temporary mechanical support-initiating IABP early in moderate AAR patients while avoiding futile interventions in those with extensive infarction, although this concept requires prospective validation.

The results of this study have several potential clinical implications. Rather than suggesting a general benefit of IABP, our data support a hypothesis that patient selection and timing may be critical determinants of its effectiveness. First, early IABP initiation could be considered in AMI-CS patients with a moderate AAR (30–65% of left ventricular mass) and hemodynamic instability. Second, patient selection appears crucial, as IABP may be most beneficial in younger patients with preserved renal function and moderate AAR. Third, if a well-defined patient population—such as those with moderate AAR and early hemodynamic compromise—can be reliably identified, IABP could be regarded as a distinct, resource-efficient MCS modality within the broader support spectrum. This differentiation would allow more rational allocation of limited healthcare resources, ensuring that IABP is deployed where it is most likely to provide meaningful clinical benefit in vulnerable populations.

## Limitations

Several key limitations must be considered when interpreting the findings. First, the retrospective, non-randomized design introduces significant selection bias and confounding by indication. Patients who received IABP were not randomly assigned but rather selected based on clinical judgment, institutional protocols, and perceived severity of shock. While statistical adjustments (e.g., multivariable regression and AIPW analysis) were applied, unmeasured confounders—such as variations in revascularization timing, adjunctive medical therapies, shock severity, or center-specific expertise—may still influence outcomes. Furthermore, the study period reflects a premodern MCS era and may not reflect contemporary practices, such as the increasing use of early revascularization strategies, adjunctive pharmacotherapies (e.g., vasopressors, inotropes, or sodium-glucose cotransporter-2 inhibitors), or use of ECMO and/or Impella. During the study period (2009–2015), European Society of Cardiology (ESC) guidelines generally recommended culprit-only percutaneous coronary intervention (PCI) as the default strategy in acute myocardial infarction (AMI) with multivessel disease. According to the 2008 and 2012 ESC STEMI and 2014 ESC/EACTS Myocardial Revascularization guidelines, complete revascularization was permissible in the presence of cardiogenic shock or persistent ischaemia. The therapeutic environment of our cohort reflects a period of evolving guidance, in which operator discretion played a substantial role in determining the extent and timing of revascularization in cardiogenic shock. Exact time-to-event data were not uniformly available across both centers, precluding pooled survival analyses using Cox regression models. Outcomes were therefore assessed at fixed time points. Treatment decisions were guided by contemporaneous recommendations; however, evolving guideline changes during the study period may have influenced IABP use and timing, with a shift from more liberal early use toward a more selective strategy following successful revascularization.

## Conclusion

This study provides hypothesis-generating insights into the association between IABP timing and outcomes in AMI-CS. Early IABP initiation during primary PCI was associated with improved in-hospital and 1-year survival, particularly in patients with moderate AAR (30–65%). Late IABP insertion was associated with a less improved in-hospital survival compared to non-rescue IABP placement; meanwhile, the 1-year survival rates were not different compared to patients without IABP treatment. This study contributes to the ongoing debate on IABP use in CS and highlights critical gaps in evidence, particularly the optimal timing and patient selection for improved long-term outcomes.

AI tools (ChatGPT 5.1) were used as a supplementary aid in drafting this manuscript. All output was rigorously reviewed and approved by the authors, who assume full responsibility for its content.

## Supplementary Information

Below is the link to the electronic supplementary material.

Supplementary file1 (DOCX 100 kb)

## Abbreviations

*AAR*: Area at risk
*AMI*: Acute myocardial infarction
*CABG*: Coronary artery bypass grafting
*CK*: Creatine kinase
*CMR*: Cardiac magnetic resonance
*CS*: Cardiogenic shock
*DM*: Diabetes mellitus
*ECG*: Electrocardiogram/electrocardiography
*GFR*: Glomerular filtration rate
*Hgb*: Hemoglobin
*IABP*: Intra-aortic balloon pump
*ICU*: Intensive care unit
*JAHA*: Journal of the American Heart Association
*LAD*: Left anterior descending (artery)
*LCX*: Left circumflex (artery)
*LMICs*: Low- and middle-income countries
*LV*: Left ventricle/left ventricular
*MACE*: Major adverse cardiovascular events
*MCS*: Mechanical circulatory support
*MI*: Myocardial infarction
*NSTE-ACS*: Non-ST-elevation acute coronary syndrome
*PAD*: Peripheral artery disease
*PCI*: Percutaneous coronary intervention
*RCT*: Randomized controlled trial
*RCA*: Right coronary artery
*SGLT2*: Sodium-glucose cotransporter 2
*STEMI*: ST-elevation myocardial infarction
*VA-ECMO*: Veno-arterial extracorporeal membrane oxygenation
