# Supplementary material for: Timing of IABP initiation and its impact on outcomes in acute myocardial infarction with cardiogenic shock: insights from a bi-center retrospective study
Source: Clin Res Cardiol. 2026 Jun 8;115(11):2005–14. doi: 10.1007/s00392-026-02951-1 (PMC13658395; doi:10.1007/s00392-026-02951-1)
Supplement: Supplementary file 1 [file 392_2026_2951_MOESM1_ESM.docx]

**Supplementary** **information**

|  | Observations |  |  |  |  |
| --- | --- | --- | --- | --- | --- |
| Treatment | Raw | Weighted |  |  |  |
|  |  |  |  |  |  |
| No-IABP (n) | 124 | 133.4 |  |  |  |
| Non-rescue-IABP (n) | 213 | 131.8 |  |  |  |
| Rescue-IABP (n) | 58 | 129.9 |  |  |  |
| Total | 395 | 395.0 |  |  |  |
|  |  |  |  |  |  |
|  |  | Standardized differences | | Variance ratio | |
|  |  | Raw | Weighted | Raw | Weighted |
|  |  |  |  |  |  |
| Non-rescue-IABP | Age | -0,505 | 0,074 | 1,141 | 0,674 |
|  | Female gender | 0,171 | 0,054 | 1,129 | 1,039 |
|  | AAR-category |  |  |  |  |
|  | 1 | 0,330 | -0,059 | 1,317 | 0,963 |
|  | 2 | 0,115 | 0,085 | 1,063 | 1,062 |
|  | PAD | 0,430 | 0,098 | 3,237 | 1,258 |
|  | Nicotin use | -0,212 | 0,076 | 0,880 | 1,055 |
|  |  |  |  |  |  |
| Rescue-IABP | Age | -0,452 | 0,085 | 0,874 | 0,530 |
|  | Female gender | 0,152 | -0,018 | 1,132 | 0,986 |
|  | AAR-category |  |  |  |  |
|  | 1 | 0,317 | -0,040 | 1,326 | 0,975 |
|  | 2 | -0,136 | 0,094 | 0,900 | 1,068 |
|  | PAD | 0,356 | 0,159 | 2,874 | 1,422 |
|  | Nicotin use | 0,005 | 0,076 | 1,011 | 1,056 |

Supplementary Table 1: Covariate balance before and after augmented inverse-probability weighting (AIPW). Standardized differences are presented for all included covariates before and after AIPW adjustment. Following weighting, excellent covariate balance was achieved, with standardized differences <0.1 for all variables. Abbreviations: IABP, intra-aortic balloon pump; AAR, area at risk; PAD, peripheral artery disease.

Supplementary Figure 1: Overlap plots demonstrating common support for propensity-based analyses. Adequate overlap between groups indicates sufficient common support, supporting the validity of the augmented inverse-probability weighting (AIPW) analysis. Abbreviation: IABP, intra-aortic balloon pump.
